# CCR7 Mediated Mimetic Dendritic Cell Vaccine Homing in Lymph Node for Head and Neck Squamous Cell Carcinoma Therapy

**DOI:** 10.1002/advs.202207017

**Published:** 2023-04-24

**Authors:** Jiabin Xu, Hong Liu, Tao Wang, Zhenfu Wen, Haolin Chen, Zeyu Yang, Liyan Li, Shan Yu, Siyong Gao, Le Yang, Kan Li, Jingyuan Li, Xiang Li, Lixin Liu, Guiqing Liao, Yongming Chen, Yujie Liang

**Affiliations:** ^1^ Hospital of Stomatology Sun Yat‐sen University Guangzhou 510030 P. R. China; ^2^ School of Materials Science and Engineering Key Laboratory for Polymeric Composite and Functional Materials of Ministry of Education Sun Yat‐sen University Guangzhou 510275 P. R. China; ^3^ Guangdong Provincial Key Laboratory of Stomatology Guangzhou 510030 P. R. China; ^4^ Institute of Stomatology Sun Yat‐sen University Guangzhou 510030 P. R. China; ^5^ School of Stomatology Xuzhou Medical University Xuzhou 221004 P. R. China; ^6^ Affiliated Stomatological Hospital of Xuzhou Medical University Xuzhou 221004 P. R. China

**Keywords:** CCR7, head and neck squamous cell carcinoma, lymph node targeting, nanovaccines, tumor‐derived exosomes

## Abstract

Immunotherapy has been recognized as one of the most promising treatment strategies for head and neck squamous cell carcinoma (HNSCC). As a pioneering trend of immunotherapy, dendritic cell (DC) vaccines have displayed the ability to prime an immune response, while the insufficient immunogenicity and low lymph node (LN) targeting efficiency, resulted in an unsubstantiated therapeutic efficacy in clinical trials. Herein, a hybrid nanovaccine (Hy‐M‐Exo) is developed via fusing tumor‐derived exosome (TEX) and dendritic cell membrane vesicle (DCMV). The hybrid nanovaccine inherited the key protein for lymphatic homing, CCR7, from DCMV and demonstrated an enhanced efficiency of LN targeting. Meanwhile, the reserved tumor antigens and endogenous danger signals in the hybrid nanovaccine activated antigen presenting cells (APCs) elicited a robust T‐cell response. Moreover, the nanovaccine Hy‐M‐Exo displayed good therapeutic efficacy in a mouse model of HNSCC. These results indicated that Hy‐M‐Exo is of high clinical value to serve as a feasible strategy for antitumor immunotherapy.

## Graphical abstract

Hybridize immunogenic tumor derived exosome (TEX) with CCR7 retained dendritic cell membrane vesicle (DCMV), and adjuvanted by MPLA, to develop a novel nanovaccine (Hy‐M‐Exo), which can target in lymph nodes via the CCR7‐CCL21 way. Hy‐M‐Exo elicits robust tumor‐specific T cells infiltrated in the tumor site, alters the tumor microenvironment, and leads to tumor regression.

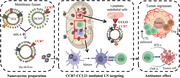

## 1 Introduction

Head and neck cancer (HNC) is the seventh most common cancer globally and occurs in the upper aerodigestive tract.^[^ ^1^ ^]^ In China, over 350000 new cases and 240 000 deaths of HNC were reported in 2016,^[^ ^2^ ^]^ of which head and neck squamous cell carcinoma (HNSCC) accounts for nearly 90%.^[^ ^3^ ^]^ Surgery is the primary treatment for HNSCC in clinical. Over the past 30 years, although curative treatment of HNSCC has made some progress, the 5‐year overall survival rate remains <50% because many patients are at advanced stages.^[^ ^3^ ^]^ Exploring novel and effective treatments for HNSCC in clinical practice is urgent.

Therapeutic vaccination has been recognized as a promising strategy for cancer therapy by activating the immune system. Particular vaccines based on subunit antigens and molecular adjuvants showed a striking effect in the application of prophylactic and therapeutic vaccines. However, as a spontaneous tumor, the HNSCC lacks definite tumor‐associated antigens (TAAs), which limits the development of antigen and adjuvant‐based vaccines. Recent studies have used the tumor cell lysates or tumor cell fragments released through chemotherapy and radiation as TAAs.^[^ ^4^, ^5^, ^6^, ^7^, ^8^ ^]^ However, the tumor cell lysates with low immunogenicity as antigens could induce immune tolerance with low antigen uptake efficiency.^[^ ^9^ ^]^ In addition, the types and amounts of antigens released by chemotherapy and radiation vary from individual to individual. Therefore, it is urgent to seek optimization strategies to prepare a new vaccine for tumors without definite TAAs. Recently, the role of exosome in tumor vaccines has been gradually emphasized. Exosomes are extracellular vesicles (EVs) with a diameter of ≈30 nm – 150 nm, produced by the fusion of the multivesicular bodies with the cell plasma membrane.^[^ ^10^ ^]^ Compared with the plasma membrane, exosomes have more different protein compositions, which enrich molecules involved in antigen presentation (such as MHC class I and II molecules, costimulatory molecules, four transmembrane proteins, heat shock proteins), and potential cell targeting molecules (such as CD11b, ICAM, four transmembrane proteins, and lactomucin).^[^ ^11^, ^12^ ^]^ Tumor‐derived exosome (TEX) is also rich with TAAs and neoantigens and could effectively deliver tumor antigens to dendritic cells (DCs) to generate specific antitumor immunity.^[^ ^13^, ^14^ ^]^ More importantly, it has been reported that TEX was superior to tumor lysate as a source of tumor antigen in antitumor efficacy.^[^ ^15^, ^16^, ^17^ ^]^ Therefore, TEX‐based tumor vaccines may have potential value in clinical applications.

It is worth noting that TEX can induce immunosuppression by suppressing the functions of T cells and NK cells and altering the numbers or activities of immune suppressor cells, including regulatory T cells (Tregs) and myeloid‐derived suppressor cells (MDSCs) in the tumor microenvironment (TME).^[^ ^18^, ^19^, ^20^ ^]^ Moreover, TEX can help cancer cells escape from immune surveillance in HNSCC, thereby promoting tumorigenesis.^[^ ^21^, ^22^ ^]^ Therefore, as a TAA, TEX should take advantage of its immunogenicity and inhibit its immunosuppressive effect.

Immune adjuvants are nonspecific immune stimulators that can enhance the immunogenicity of antigens and broke the immune tolerance in tumor therapy.^[^ ^23^ ^]^ Moreover, adjuvants can promote antigen uptake and presentation by antigen presenting cells and reduce the amount of antigen used.^[^ ^24^ ^]^ In this study, we applied monophosphoryl lipid A (MPLA), a Toll‐like‐receptor 4 (TLR4) adjuvant,^[^ ^25^ ^]^ synergized with HNSCC‐derived TEX. The nanoparticular vaccine has the advantage of targeting the lymph nodes (LNs) via lymph draining,^[^ ^26^, ^27^ ^]^ inducing a robust immune response and reducing the adjuvant's systemic toxicity.^[^ ^28^, ^29^ ^]^ To further enhance targeting LNs, we hybridized the TEX with dendritic cell membrane vesicle (DCMV) and adjuvanted by MPLA to form a novel nanovaccine. In detail, CC‐chemokine receptor 7 (CCR7) is highly expressed on the membrane of mature DCs, and mediates mature DCs’ lymphoid homing through binding with CCL21/19 ligands on the surface of lymphatic endothelial cells.^[^ ^30^ ^]^ We borrowed the CCR7‐CCL21/19 axis by fusing the TEX with the membrane vesicle derived from mature DCs, so that the hybrid vesicles can actively migrate to LNs via an “active targeting” pathway.

**Figure 1:**
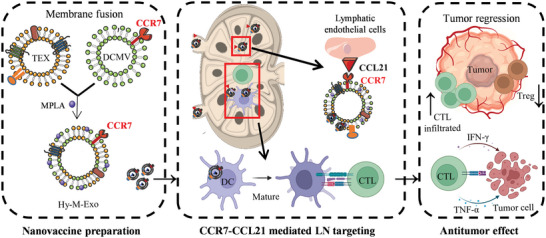
Schematic illustration for the mechanism of T cell‐based immunotherapy elicited by Hy‐M‐Exo for tumor inhibition.

Thus, we constructed hybrid nanovesicles (named Hy‐M‐Exo) consisting of TEX and DCMV with MPLA for immunotherapy (**Figure** **1**). The obtained Hy‐M‐Exo possessed similar proteins with DCs (such as CD86 and CCR7) and demonstrated a significant effect to target the paracortex of the LNs where the DCs and T cells were abundant. Hy‐M‐Exo elicited a robust DC activation and T‐cell response in LN, which showed potent inhibition of HNSCC in mice. In addition, we found that the Hy‐M‐Exo could down‐regulate the Tregs and thus reduces the immune tolerance.^[^ ^31^, ^32^ ^]^ To sum up, we propose that the strategy using the CCR7‐CCL21/19 mediated pathway for delivering drugs to LN is promising.

## 2 Results and Discussions

### 2.1 Fabrication and Characterization of Hy‐M‐Exo

**Figure 2:**
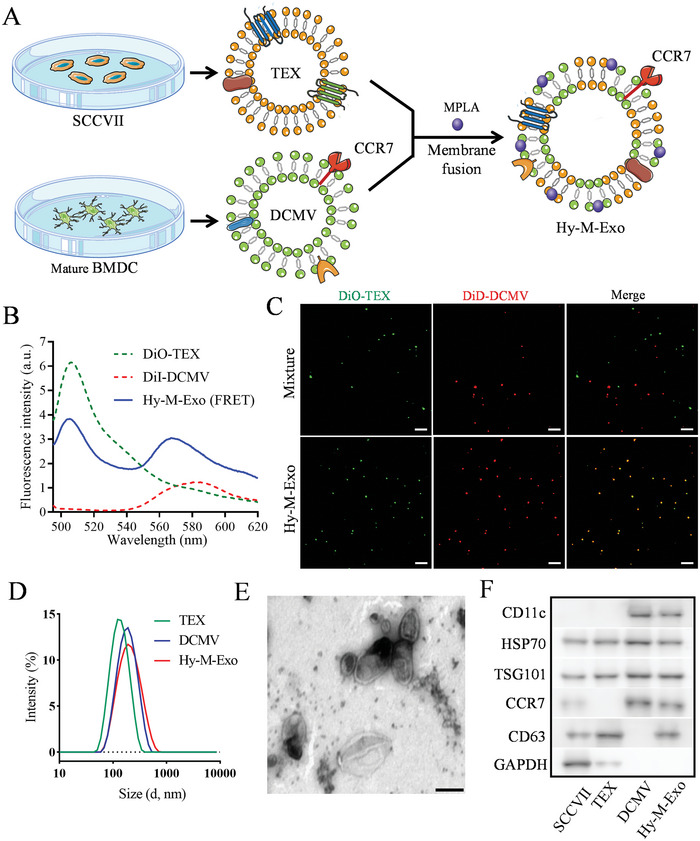
Preparation and characterization of Hy‐M‐Exo. A) Schematic illustration for the preparation of Hy‐M‐Exo, which was fabricated by TEX fusion with CCR7‐retained DCMV. B) Fluorescence resonance energy transfer (FRET) of DiO‐labeled TEX and DiI‐labeled DCMV (excitation = 484 nm, emission = 495–620 nm). C) CLSM images of a physical mixture (TEX with DCMV) (mixture) and Hy‐M‐Exo (fusion) (green, DiO‐labeled TEX; red, DiD‐labeled DCMV, scale bar: 5 µm) D) Hydrodynamic size of Hy‐M‐Exo measured by dynamic light scattering. E) Representative TEM image of Hy‐M‐Exo stained with uranyl acetate, scale bar: 100 nm. F) Specific protein markers of TEX, DCMV, and Hy‐M‐Exo are characterized by WB.

TEX (TEX was derived from SCCVII cells if not specially mentioned) was isolated by ultracentrifugation from the SCCVII cell culture supernatants. DCMV was harvested by serial extrusion of TEX‐treated bone marrow‐derived dendritic cells (BMDCs) and stepwise centrifugation as previously described (**Figure** **2**A).^[^ ^33^ ^]^ Then, the Hy‐M‐Exo was fabricated by extruding the mixture of TEX, DCMV, and MPLA. The Forster resonance energy transfer (FRET) assay was adopted to verify the membrane fusion of TEX and DCMV. The TEX and DCMV were labeled with 3,3′‐dioctadecyloxacarbocyanine perchlorate (DiO) and 1,1′‐dioctadecyl‐3,3,3′,3′‐tetramethylindocarbocyanine perchlorate (DiI), respectively. In Figure 2B, Hy‐M‐Exo demonstrated a noticeable FRET effect, indicating that the TEX membrane was fused into the DCMV membrane (Figure 2B). Furthermore, TEX and DCMV were labeled with DiO (green) and 1,1′‐dioctadecyl‐3,3,3′,3′tetramethylindodicarbocyanine‐4‐chlorobenzenesulfonate salt (DiD, red), respectively, and then imaged using a confocal laser scanning microscope (CLSM). The images of CLSM demonstrated that the green fluorescence of DiO‐TEX and the red fluorescence of DiD‐DCMV merged entirely, indicating the fusion of the TEX membrane and DCMV membrane (Figure 2C).

The dynamic light scattering (DLS) showed that Hy‐M‐Exo had a hydrodynamic size at 176.6 nm with PDI at 0.204, while TEX and DCMV were 124.0 nm (PDI = 0.133) and 160.1 nm (PDI = 0.179, Figure 2D, Figure S1A, Supporting Information). The transmission electron microscopy (TEM) results demonstrated that Hy‐M‐Exo had a typical round‐shaped morphology of membrane vesicle (Figure 2E). The surface zeta potential of TEX, DCMV, and Hy‐M‐Exo was −12.9 ± 0.78 mV, −11.73 ± 0.41 mV, and −12.63 ± 1.20 mV, which corresponded to the zeta potential of the cell membrane (Figure S1B, Supporting Information). In addition, the MPLA was loaded with Hy‐M‐Exo via hydrophobic sorption, and the loading efficiency of MPLA was 42.3 ±  6.6%.

Western blot (WB) analysis and Coomassie blue staining were used to investigate further whether the fabrication process of Hy‐M‐Exo would affect the critical proteins’ activity in TEX (CD63, TSG101, and HSP70 in Figure 2F) and DCMV (CCR7 and CD11c in Figure S2A,B, Supporting Information). As shown in Figure 2F and Figure S1C, Supporting Information, the WB analysis and Coomassie confirmed the marker proteins of TEX and DCMV, especially, the CCR7 in Hy‐M‐Exo showed high activity.

Overall, the membranes of TEX and DCMV were successfully fused, and a stable hybrid vesicle named Hy‐M‐Exo was affirmed, which ensured the feasibility of subsequent experiments. In particular, possessing membrane protein CCR7 derived from mature DCs may facilitate Hy‐M‐Exo with active LN targeting.

### 2.2 Enhancement of LN Targeting by Hy‐M‐Exo

**Figure 3:**
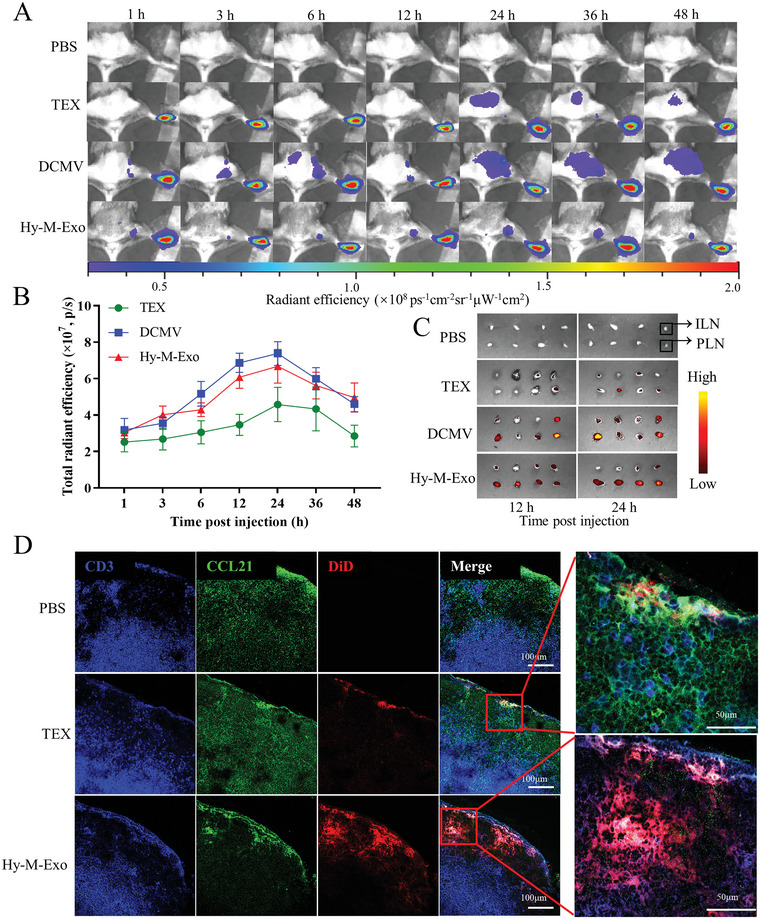
Hy‐M‐Exo targeting lymph nodes. C3H mice were injected with DiR‐labeled nanovesicles. A) Fluorescence of the injection site was observed with IVIS imaging at 1, 3, 6, 12, 24, 36, and 48 h post‐injection. B) Total radiant efficiency of mice at different time post‐injection. Data were means ±  standard error of mean deviation (SEM) (*n* = 4). C) Popliteal and inguinal LNs were excised and analyzed with IVIS imaging at 12 and 24 h post‐injection. D) Popliteal LNs were excised, cryostat‐sectioned, stained, and observed under CLSM at 6 h post‐injection.

To verify whether CCR7‐retained Hy‐M‐Exo could enhance the LN targeting, various nanovesicles labeled with 1,1‐dioctadecyl‐3,3,3,3‐tetramethylindotricarbocyaine iodide (DiR) was injected into the footpad of mice. As **Figure** **3**A,B showed, both DCMV and Hy‐M‐Exo displayed a considerable accumulation in popliteal LNs (PLN) at 1 h post‐injection and peaked at 24 h, respectively. Whereas TEX showed relatively weaker signals in PLNs during this period of post‐injection. Subsequently, PLNs and inguinal LNs (ILNs) were harvested and imaged at 12 h and 24 h post‐injection (Figure 3C). The ex vivo images of LNs showed that more DCMV and Hy‐M‐Exo had migrated to PLNs which is consisted with the results in vivo. More exciting, Hy‐M‐Exo also migrated and accumulated in ILNs. It is believed that smaller nanoparticles may be transported to LNs more efficiently than larger ones.^[^ ^34^ ^]^ Hy‐M‐Exo was ≈50 nm bigger in diameter than TEX (176.6 nm vs 124.0 nm). However, Hy‐M‐Exo more efficiently migrated and accumulated into draining LNs (DLNs), which contributed to the CCR7 on the surface of Hy‐M‐Exo membrane for homing to LNs.^[^ ^35^ ^]^

Moreover, the immunofluorescence of PLNs was observed under CLSM to further verify the distribution of Hy‐M‐Exo in PLNs at 6 h post‐injection. As shown in Figure 3D, the CCR7‐embedded Hy‐M‐Exo (red) colocalized obviously with CCL21 (green) expressed in the lymphatic vessels. In addition, CCR7‐CCL21 axis mediated the Hy‐M‐Exo migrated to the paracortical area of the LN, where the CD3 (blue) resides. By contrast, the fluorescence signal of TEX was observed only in the subcapsular sinus. These results demonstrated that Hy‐M‐Exo was superior to TEX in LN targeting due to the presence of CCR7.

### 2.3 Promotion of Internalization and Activation of APCs by Hy‐M‐Exo

**Figure 4:**
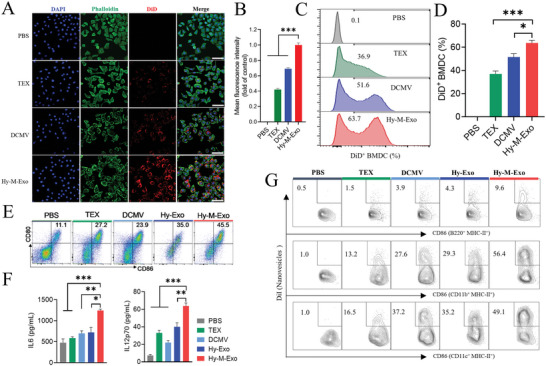
Promotion of internalization and activation of APCs by Hy‐M‐Exo. A) Representative CLSM images and B) mean fluorescence intensity (MFI) of DC2.4 cells after incubation with PBS, DiD‐labeled TEX, DCMV, or Hy‐M‐Exo for 4 h. Blue represented nuclei, green represented cytoskeleton, and red represented nanovesicles, scale bar: 50 µm. The MFI of Hy‐M‐Exo group was used as the control. C,D) Cellular uptake of DiD‐labeled nanovesicles by BMDCs was measured by flow cytometry. E) Representation flow cytometry plots of mature BMDCs (CD80^+^CD86^+^) after incubation with various nanovesicles for 24 h. F) Levels of cytokines IL6 and IL12p70 in supernatants of cultured BMDCs were detected by ELISA. G) Representation flow cytometry diagrams of mature APCs in draining LNs. C3H mice were injected with DiI‐labeled nanovesicles. Popliteal and inguinal LNs were harvested for flow cytometry at 12 h post‐injection. Data were presented as the mean ±  SEM. (*n* = 5). Statistical analysis was performed using an unpaired two‐tailed Student's *t*‐test. *p < 0.05, **p < 0.01, ***p < 0.001.

Vaccines internalized effectively by the APCs is crucial for antigen‐presenting and following to activate the innate immune response.^[^ ^36^ ^]^ Here, the internalization of different formulations of vaccines was first evaluated in DC2.4 cells by CLSM. The results showed that cells incubated with Hy‐M‐Exo (red) displayed significantly higher efficiency in nanovesicle uptake (**Figure** **4**A,B). Subsequently, flow cytometry analysis was used further to evaluate the internalization of different vaccines in BMDCs. As Figure 4C,D shows the internalization of Hy‐M‐Exo increased by 1.7‐fold, compared with TEX.

Cells appear to take up EVs by a variety of endocytic pathways, including clathrin‐dependent endocytosis, clathrin‐independent pathways such as caveolin‐mediated endocytosis, macropinocytosis, phagocytosis, and membrane fusion.^[^ ^37^ ^]^ To figure out the underlying mechanism of the efficient internalization of Hy‐M‐Exo, a series of experiments were carried out. First, to test whether the uptake is an active process or passive membrane fusion, DC2.4 cells were incubated with DiD‐labeled nanovesicles at 37 °C or 4 °C for 4 h. Figure S3, Supporting Information, showed that the internalization of TEX or DCMV was nearly completely inhibited at 4 °C. While Hy‐M‐Exo, suffering significantly inhibited, still showed certain internalization. These results indicate that the internalization of TEX or DCMV is mediated by active endocytic processes and is energy‐dependent, while the uptake of Hy‐M‐Exo is partly mediated by a passive membrane fusion route.

To further identify the internalization pathways, inhibitors of specific uptake routes were employed, including clathrin‐mediated endocytosis inhibitor chlorpromazine (CPZ), caveolae‐mediated endocytosis inhibitor genistein, macropinocytosis inhibitor LY294002, phagocytosis blocker wortmannin, and membrane fusion inhibitor omeprazole.^[^ ^37^, ^38^, ^39^ ^]^ Figure S4A,B, Supporting Information, indicated that the uptake of TEX was mediated by caveolin‐mediated endocytosis and micropinocytosis, and that of DCMV was mediated mainly by clathrin‐mediated endocytosis and partly by micropinocytosis, while the internalization of Hy‐M‐Exo was a heterogeneous process, mediated by the synergy of multiple pathways, including caveolin‐mediated endocytosis, micropinocytosis, membrane fusion, and clathrin‐mediated endocytosis.

Together with all the evidence, the following reasons might contribute to the higher uptake efficiency of Hy‐M‐Exo by DCs. Firstly, generated by membrane fusion of TEX and DCMV, Hy‐M‐Exo can be more active in cell membrane fusion (Figure S3, Supporting Information). Secondly, Multiple uptake pathways synergistically participate in the internalization of Hy‐M‐Exo (Figure S4A,B, Supporting Information). What is more, the attached MPLA could not only mediate TLR4‐targeting to DCs (Figure S4C, Supporting Information), but also be capable to induce activation of clathrin‐dependent or clathrin‐independent endocytosis.^[^ ^40^ ^]^

The maturation of BMDCs by vaccines post‐internalization is critical for the subsequent antigen presentation process. In this study, the expression of costimulatory molecules CD80 and CD86 was analyzed to estimate the maturation of BMDCs. As Figure 4E and Figure S5A, Supporting Information, shows, Hy‐M‐Exo stimulated ≈2.2 folds of CD80 and CD86 expressing to TEX, ≈1.8 folds to DCMV, and ≈1.7 folds to Hy‐Exo, respectively. Furthermore, we checked the proinflammatory cytokine, IL6, and the Th1 polarizing cytokine, IL12p70, secreted by BMDCs during maturation. Compared with other groups, Hy‐M‐Exo resulted in more excretion of IL6 and IL12p70 (Figure 4F), and the preexisting innate immune responses indicated that Hy‐M‐Exo could stimulate and regulate adaptive immune responses potentially.^[^ ^41^ ^]^ These results suggested that Hy‐M‐Exo showed more potent BMDC responses to promote subsequent adaptive immunity. This was probably owing to the MPLA and endogenous danger signals (such as HSP70) retained in Hy‐M‐Exo synergistically augmented the activation of BMDCs.^[^ ^42^ ^]^

Moreover, the Hy‐M‐Exo function on the professional APCs in LNs was comprehensively characterized by detecting MHC‐II expression. DiI‐labeled various vaccines were injected into the footpad of mice, and the maturation of B cells, macrophages, and DCs internalized with vaccines in PLNs and ILNs were evaluated at 24 h post‐injection by flow cytometry. As Figure 4G and Figure S5B‐D, Supporting Information, shows Hy‐M‐Exo activated significant maturation of B cells (CD86^+^MHC‐II^+^B220^+^), macrophages (CD86^+^MHC‐II^+^CD11b^+^), and DCs (CD86^+^MHC‐II^+^CD11c^+^).

The robust innate immune response elicited by Hy‐M‐Exo in vitro and in vivo implied that the subsequent T‐cell immune responses were stimulated.

### 2.4 Elicitation of Robust T‐Cell Response by Hy‐M‐Exo In Vivo

**Figure 5:**
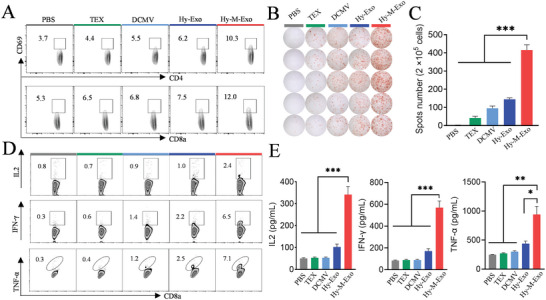
Elicitation of robust T‐Cell response by Hy‐M‐Exo in vivo. C3H mice were injected with 3 doses of various nanovesicles. Mice were sacrificed 3 days after the last vaccination. The peripheral blood, draining LNs, and spleens were harvested for analysis. A) Frequency of CD69^+^CD4^+^ and CD69^+^CD8^+^ T cells in dLNs. B) ELISPOT assay and C) quantitative analysis of IFN‐*γ* specific splenocytes after restimulation. D) Frequency of IL2^+^CD8^+^, IFN‐*γ* ^+^CD8^+^, and TNF‐*α* ^+^CD8^+^ restimulated spleen cells. E) Cytokines IL2, IFN‐*γ*, and TNF‐*α* levels in peripheral serum of immunized mice detected by ELISA. Representative data were expressed as mean ±  SEM. (*n* = 5). One‐way ANOVA with Dunnett's post hoc analysis was used to calculate statistical significance. *p < 0.05, **p < 0.01, ***p < 0.001.

Encouraged by the deep fused into LN of Hy‐M‐Exo and high APC activation efficiency, we examined the T cell immune stimulation property of Hy‐M‐Exo in vivo. The mice were vaccinated with PBS, TEX, DCMV, Hy‐Exo, and Hy‐M‐Exo. The activation of CD69^+^CD4^+^ and CD69^+^CD8^+^ T cells in draining LNs was detected by flow cytometry three days after the last vaccination. As shown in **Figure** **5**A and Figure S6B,C, Supporting Information, the Hy‐M‐Exo achieved ≈2.8 folds, ≈2.3 folds, ≈1.9 folds, and ≈1.7 folds CD69^+^CD4^+^ and ≈2.3 folds, ≈1.9 folds, ≈1.8 folds and ≈1.6 folds CD69^+^CD8^+^ higher than PBS, TEX, DCMV, and Hy‐Exo treated mice. In addition, spleens were also harvested for flow cytometry analysis and ELISPOT assay. Flow cytometry results showed that Hy‐M‐Exo elicited more CD69^+^CD4^+^ (18.8%) and CD69^+^CD8^+^ (38.1%) T cells (Figure S6D‐F, Supporting Information), and it was more exciting that Hy‐M‐Exo‐treated mice produced significantly more IL2^+^CD8^+^ (2.4%), IFN‐*γ* ^+^CD8^+^ (6.5%), and TNF‐*α* ^+^CD8^+^ (7.1%) effector T cells (Teff) than other groups (Figure 5D, and Figure S6G, Supporting Information). These results were confirmed by the robust IFN‐*γ* ELISPOT response of Hy‐M‐Exo. After restimulation, there were 2.5‐fold more spots in the Hy‐M‐Exo group than in the Hy‐Exo group, which were much more than that in other groups (Figure 5B,C). Moreover, immunostimulatory cytokines IL2, IFN‐*γ*, and TNF‐*α* were significantly enhanced in the peripheral blood of mice treated with Hy‐M‐Exo (Figure 5E). These results indicated that Hy‐M‐Exo could efficiently induce LN to systemic T‐cell activation. Concerning HNSCC, CD8^+^ T cells are believed to play a critical role in tumor suppression as effector cells.^[^ ^43^ ^]^

### 2.5 Inhibition of Tumor Growth by Hy‐M‐Exo In Vivo

**Figure 6:**
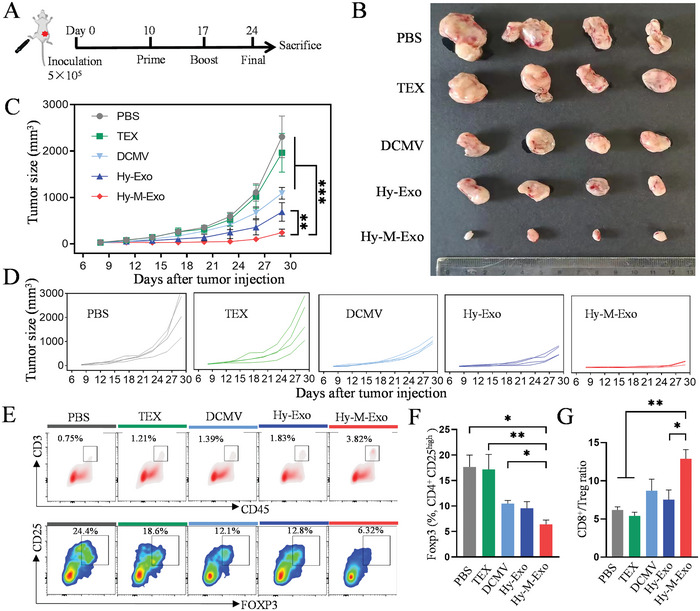
Inhibition of tumor growth by Hy‐M‐Exo in vivo. A) Schematic diagram of the therapeutic study in C3H mice. B) The image of the tumor masses. C) Average and D) individual tumor growth curves of tumor‐bearing mice vaccinated with various nanovesicles. Mice were sacrificed when the tumor size of the PBS group exceeded 2000 mm^3^, and peripheral blood and tumor masses were collected to analyze immune responses. E) Representative flow cytometry plots of CD3^+^CD45^+^ TILs and FOXP3^+^CD25^high^CD4^+^ Tregs in the tumor masses of mice. Quantitative analysis of F) FOXP3^+^CD25^high^CD4^+^ Tregs and G) ratio of CD8^+^ Teff to Treg in tumor masses. Data were shown as mean ±  SEM (*n* = 4). Statistical analysis was performed using (C) two‐way ANOVA with Tukey's multiple comparisons test and (F, G) unpaired two‐tailed Student's *t*‐test. *p < 0.05, **p < 0.01, ***p < 0.001.

The high efficacy of Hy‐M‐Exo to elicit T‐cell response prompted us to explore whether the activated immune response can inhibit tumor growth in tumor‐bearing mice. The HNSCC tumor model was established by injection of SCCVII cells into the right flank of the mouse subcutaneously. When the tumor volume approached nearly 50 mm^3^, different vaccines were administered subcutaneously into mice's rear footpad once a week for three weeks (**Figure** **6**A). Like PBS control, TEX did not show any inhibition of tumor growth. Among DCMV, Hy‐Exo, and Hy‐M‐Exo groups that suppressed tumor growth, Hy‐M‐Exo demonstrated the maximum therapeutic effect and at the end of monitoring the tumor volume decreased to 200 mm^3^, in contrast to that of the PBS group to 2000 mm^3^ (Figure 6B–D). Immunosuppressive effect of TEX in TME made it difficult to generate anti‐tumor immunity without modification. DCMV obtained from BMDCs stimulated by TEX and Hy‐Exo showed certain therapeutic effects, mainly due to the antigen on TEX being successfully presented on the DCMV. Moreover, with the adjuvant MPLA amplifying immune stimulation, Hy‐M‐Exo displayed robust tumor inhibition.

To find out the underlying mechanism of antitumor efficacy by Hy‐M‐Exo, we further analyzed the immune profiles in tumor masses. It is agreed in reports that tumor‐infiltrating lymphocytes (TILs) are associated with a favorable prognosis for survival of HNSCC patients.^[^ ^43^ ^]^ Flow cytometry results showed that the CD3^+^CD45^+^ TILs in mice treated with Hy‐M‐Exo were the highest among all groups. In addition, there was a significantly lower percentage of FOXP3^+^CD25^high^CD4^+^ tumor‐infiltrating Tregs of Hy‐M‐Exo–vaccinated mice compared with mice of other groups (Figure 6E). The ratio of CD8^+^ Teff to Treg in the Hy‐M‐Exo group was increased ≈2.1, ≈2.4, ≈1.5, and ≈1.7‐fold compared with the PBS, TEX, DCMV, and Hy‐Exo groups (Figure 6F,G). Herein, LNs orchestrated the innate with the adaptive immune response of the antigen. Moreover, the Hy‐M‐Exo targeted in LNs could elicit a local to systemic immune response. In other words, the adjuvant in the Hy‐M‐Exo stimulated a robust innate immune response and thereafter elicit the potent specific immune response to the antigen. Then, the activated T cells migrated from the LNs to the tumor sites to alter the TME from inhibiting to killing.

Together, these results validated the immunostimulation effect of Hy‐M‐Exo in tumor‐bearing mice, thus obtaining a favorable antitumor response in an HNSCC model. Regrettably, Hy‐M‐Exo monotherapy cannot completely eliminate the tumor, and thus we believe that tumor vaccine should act as a complementary treatment in combination therapy.^[^ ^44^ ^]^

### 2.6 Biosafety Assessment of Hy‐M‐Exo

Safety is of top importance for a vaccine. Theoretically, generated by the cell membrane and Food and Drug Administration‐approved vaccine adjuvant MPLA, Hy‐M‐Exo should be biologically safe for organisms. Our study testified the biosafety of Hy‐M‐Exo both in vitro and in vivo. First, DC2.4 or L929 cells were incubated with different concentrations of Hy‐M‐Exo for 24 h, and then Cell Counting Kit‐8 (CCK‐8) was applied to evaluate cell viability. As Figure S7A,B, Supporting Information, shows the viability of DC2.4 or L929 cells remained above 90% without being influenced by increased concentration. Hemolysis assay also verified the in vitro biocompatibility as no significant hemolysis was found in the presence of Hy‐M‐Exo (Figure S7C, Supporting Information). The body weight of Hy‐M‐Exo treated mice remained stable and comparable to that of the mice in other groups during the course of treatment (Figure S7D, Supporting Information). Moreover, histological analysis with hematoxylin and eosin (H&E)–stained sections of major organs demonstrated that no obvious damages were observed, demonstrating good in vivo biosafety of Hy‐M‐Exo (Figure S7E, Supporting Information).

## 3 Conclusion

In summary, the advanced hybrid nanovesicles Hy‐M‐Exo via fusing immunogenic TEX with functional DCMV were successfully developed in this study. As a carrier and vaccine, the Hy‐M‐Exo was endowed with the LN homing property like DCs and TAAs from TEX. The CCR7 on DCMV could mediate the vaccine migrating to LNs via an active pathway instead of the conventional passive way, which broke the limitation of relying on small‐size nanoparticles (<100 nm) to target LNs through lymphatic drainage. Moreover, TEX was rich in the TAAs and tumor‐borne species, and along with the MPLA, TEX showed high immunogenicity. Hy‐M‐Exo efficiently migrated to LNs and in turn, elicited a robust innate immune response and T‐cell activation, and amplified tumor growth inhibition in a mouse HNSCC model. The hybrid nanovaccine has potential clinical value to serve as a feasible strategy for antitumor immunotherapy.

We could make further efforts for clinical transformation of this hybrid nanovaccine Hy‐M‐Exo. 1) TEX could be obtained through surgically resected tumors, and DCs could be differentiated from peripheral blood and then expanded in vitro. However, efficient isolation of tumor cells or DCs and large‐scale TEX or DC manufacturing must be optimized further. 2) The hybrid vesicles can load various vaccine adjuvants for enhanced antitumor efficacy. 3) Surface modification of Hy‐M‐Exo could improve accumulation and retention in the tumor, leading to a more efficient tumor‐specific immune response. 4) Hy‐M‐Exo could be combined with other therapeutic therapies, such as radiotherapy, a checkpoint inhibitor, or photothermal therapy, to pursue a synergistic antitumor effect. 5) Fusing other cell‐derived membrane vesicles (T cells, B cells, or macrophages) with TEX may also construct functional hybrid nanovesicles for tumor management. 6) More in‐depth research is needed to meet various challenges in clinical transformation, such as the dose in monotherapy or combination therapy, mass manufacturing, and long‐term storage.

## 4 Experimental Section

### Materials and Reagents

Actin‐Tracker Green (catalog: C1033), Bicinchoninic acid (BCA) kit (catalog: P0012S), Bovine Serum Albumin (catalog: ST023), Coomassie Blue Staining Solution (catalog: ST030), DAPI Staining Solution (catalog: C1005), DiO (catalog: C1038), DiI (catalog: C1036), DiD (catalog: C1039), Phenylmethylsulfonyl fluoride (PMSF, catalog: ST506) and Protease Inhibitor Cocktail (catalog: P1005) was purchased from Beyotime Biotechnology (Haimen, China). CCK‐8 (catalog: CK04) was purchased from Dojindo (Kumamoto, Japan). DiR Iodide was purchased from Yeasen Biotechnology (Shanghai, China). Murine recombinant granulocytemacrophage colony‐stimulating factor (GM‐CSF) and murine recombinant IL4 was purchased from Novoprotein (Shanghai, China). Antibodies for western blot were purchased from Abcam (Cambridge, England). Rabbit anti‐CCL21 antibody was purchased from Bioss (Beijing, China). Anti‐TLR4 monoclonal antibody was purchased from Proteintech (Chicago, USA). Anti‐mouse CD11c‐PE, anti‐mouse CD16/32, anti‐mouse CD80‐FITC, anti‐mouse CD86‐APC, anti‐mouse CD3*ε*‐AF488, anti‐mouse IFN‐*γ*‐PE, anti‐mouse IL2‐APC, anti‐mouse TNF‐*α*‐APC/Cy7, Zombie Violet™ Fixable Viability Kit, anti‐mouse CD11b‐Brilliant Violet 510, anti‐mouse CD4‐PE/Cy7, anti‐mouse CD69‐APC/Cy7, anti‐mouse B220‐PE/Cy7, anti‐mouse Gr‐1‐AF480, anti‐mouse CD25‐PE, anti‐mouse CD8*α*‐Alexa Fluor 700, anti‐mouse CD45‐Percp, anti‐mouse I‐A/I‐E‐ Brilliant Violet 605™, anti‐mouse CD163‐APC, anti‐mouse‐CD45−FITC, anti‐mouse F4/80‐PE, and anti‐mouse CD4‐FITC, anti‐mouse Foxp3‐AF647, and anti‐mouse CD3*ε*‐AF488 were purchased from Biolegend (San Diego, USA). Mouse IL2 ELISA kit (catalog: 1 210 202), mouse IL1*β* ELISA kit (catalog: 1 210 122), mouse IL12p70 ELISA kit (catalog: 1 211 202), mouse TNF‐*α* ELISA kit (catalog: 1 117 202), mouse IFN‐*γ* ELISA kit (catalog: 1 210 002), and mouse IFN‐*γ* ELISPOT kit (catalog: 2 210 002) was purchased from Dakewe Biotech (Beijing, China). Chlorpromazine, genistein, LY294002, wortmannin and omeprazole were purchased from MedChemExpress (New Jersey, USA). All cell culture medium including Roswell Park Memorial Institute (RPMI) 1640 and Dulbeccos modified Eagle medium (DMEM) were purchased from KeyGEN BioTECH (Jiangsu, China). Fetal bovine serum (FBS), penicillin–streptomycin, and trypsin–ethylenediaminetetraacetic acid (0.25%) were purchased from Gibco (Thermo Fisher Scientific, MA, USA).

### Cell Culture

Murine oral squamous cell carcinoma cell line SCCVII was a kind gift from Peking University School and Hospital of Stomatology. DC2.4 cells and L929 cells were purchased from KeyGEN BioTECH (Jiangsu, China). Cell lines were cultured in DMEM medium supplemented with 10% FBS and 1% penicillin–streptomycin at 37 °C in 5% CO_2_ for no more than ten passages.

### Animals

All SPF animals were housed in the Laboratory Animal Center of Sun Yat‐sen University (SYSU). The study complied with all principles from the Institutional Animal Care and Use Committee, SYSU (SYSU‐IACUC‐2021‐000363).

### Preparation of TEX

Cells were cultured (48 h) in a serum‐free medium. Cleared supernatants (10 min, 300 g, 2 × 15 min, 800 g, 30 min, 10 000 g) were ultracentrifuged (90 min, 100 000 g) and washed (PBS, 90 min, 100 000 g). The resuspended vesicle was purified by discontinuous sucrose gradient centrifugation.

### Preparation of BMDC

As in the previous description, mouse BMDCs from the femurs and tibias were generated. All primary cells were growing with the complete RPMI‐1640 medium supplemented with recombinant GM‐CSF (20 ng mL^−1^) and IL4 (5 ng mL^−1^) after lysis of red blood cells (RBCs). Immature BMDCs were generated after 7 days. Mature BMDCs were harvested after stimulation with TEX (50 µg mL^−1^) for 24 h on day 9.

### Preparation of DCMV

Derivation of the cell membrane of BMDCs was adapted from a previously published method with some modifications.^[^ ^45^ ^]^ Briefly, 5 × 10^7^ mature BMDCs were resuspended in 2 mL of hypotonic using buffer containing Protease Inhibitor Cocktail on ice for 30 min. Cells were enucleated using a handheld glass homogenizer (50 passes on ice), and then centrifuged at 800 g for 5 min. The supernatant was collected and the pellet was resuspended again. Homogenization and centrifugation were repeated three times at 4 °C. The supernatants were pooled and then centrifuged at 20 000 g for 20 min at 4 °C. The enriched membranes were washed twice with PBS at 4 °C.

### Preparation and Characterization of Hy‐M‐Exo

The TEX, DCMV, and MPLA were mixed at a ratio of 5: 5: 1 (TEX’ protein: DCMV’ protein: MPLA). After incubating in a 45°C water bath for 30 min, the mixture was then vortexed and sonicated for 5 min. Subsequently, the suspension was physically extruded 11 times through 0.8 µm, 0.4 µm, and 0.2 µm polycarbonate membrane, using a miniextruder (Avanti, USA), to obtain uniform Hy‐M‐Exo. Free MPLA was purified using Nanosep (MWCO = 300 K). To quantify the amount of MPLA within Hy‐M‐Exo, a LAL assay was performed, according to the manufacturer's protocol. The morphology of Hy‐M‐Exo was observed through TEM. First, Hy‐M‐Exo was diluted in PBS. Each sample was deposited onto an EM grid and observed under a TEM instrument (JEOL, Akishima, Japan) at 100 kV. Zetasize Nano ZS (Malvern Instruments, Malvern, UK) was used to test the size distribution and the zeta potential.

### Preparation of Hy‐Exo

TEX was mixed with DCMV at a protein ratio of 1: 1. After incubation, sonicated and extruded (same membrane fusion process as Hy‐M‐Exo), Hy‐Exo was fabricated. Hy‐Exo lacks MPLA compared with Hy‐M‐Exo.

### Verification of Membrane Fusion

To evaluate whether TEX was incorporated into DCMV, firstly, FRET assay was used to prove the membrane fusion by labeling TEX with lipophilic fluorescent dye DiO (excitation/emission = 484/501 nm) and DCMV with DiI (excitation/emission = 549/565 nm). Then, DiO‐labeled TEX was added to DiI‐labeled DCMV at a ratio of 1:1 for sonication and hydration, and then extruded through 0.2 µm polycarbonate membrane filters with a miniextruder. The emission wavelength was recorded from 495 to 620 nm, at the excitation wavelength of 484 nm. Subsequently, TEX and DCMV were labeled with DiO (green) or DiD (red), respectively. After sonication and hydration, the labeled TEX and DCMV were then extruded through 0.2 µm polycarbonate membranes. The fusion of TEX and DCMV was imaged under Leica TCS SP8 CLSM (Wetzlar, Germany).

### Protein Analysis of TEX, DCMV, and Hy‐M‐Exo

The SCCVII cells, TEX, BMDCs, DCMV, and Hy‐M‐Exo were first lysed with RIPA buffer to obtain proteins. After denaturation, proteins were then loaded into 10% sodium dodecyl sulfate−polyacrylamide gel electrophoresis (SDS−PAGE), transferred onto the polyvinylidene fluoride (PVDF) membrane, and probed with certain primary antibodies and secondary antibodies. Finally, an ECL chemiluminescence detection kit (Beyotime Biotech, Shanghai, China) was applied to detect proteins.

### In Vitro Cellular Uptake of Hy‐M‐Exo

The cellular uptake of Hy‐M‐Exo was measured through CLSM and flow cytometry assay (Life Technology, USA). Briefly, 1 × 10^5^ DC2.4 cells were cultured in a confocal microscopy dish overnight. Then DC2.4 cells were incubated with DiD‐labeled TEX, DCMV, or Hy‐M‐Exo in fresh DMEM at 37 °C or 4 °C for 4 h. For the uptake inhibition assay, cells were pretreated with different kinds of inhibitors for 30 min before being incubated with DiD‐labeled nanovesicles. Then cells were fixed under 4% paraformaldehyde for 10 min and washed with cold PBS containing 0.1% Triton X‐100 three times. After the nucleus was stained by DAPI and the cytoskeleton was stained by phalloidin‐FITC, cells were subjected to CLSM. As for the flow cytometry analysis, BMDCs (1 × 10^5^) were placed in a 24‐well plate and incubated with DiD‐labeled TEX, DCMV, or Hy‐M‐Exo for 4 h. Then, cells were harvested and resuspended with 400 µL of precooled PBS for flow cytometry detection.

### Induced Maturation of BMDCs by Hy‐M‐Exo

BMDCs were cultured as mentioned above. On day 7, TEX (50 µg mL^−1^), DCMV (50 µg mL^−1^), or Hy‐M‐Exo (50 µg mL^−1^, MPLA 5 µg mL^−1^) were added to pulse immature BMDCs. After 24 h, cells were collected and then blocked with anti‐mouse CD16/32 antibody in 1% BSA‐PBS buffer for 20 min at 4 °C. Subsequently, cells were incubated with PE‐anti‐CD11c antibody, FITC‐anti‐CD80 antibody, and APC‐anti‐CD86 antibody for 30 min at 4 °C. After being washed twice, cells were resuspended in 300 µL 1% BSA‐PBS buffer for flow cytometry analysis. Meanwhile, the supernatant of BMDCs was collected for the detection of immunostimulatory cytokines (IL6 and IL12p70) by the ELISA kit (Dakewe Biotech, Shenzhen, China).

### LN imaging Assay

TEX, DCMV, and Hy‐M‐Exo were labeled with DiR. Mice were injected with 0.1 mL of PBS, TEX (50 µg protein), DCMV (50 µg protein), or Hy‐M‐Exo (5 µg MPLA, and 50 µg protein) in the footpad of mice's left hind leg. Afterward, mice were imaged under IVIS Lumina X5 system (PerkinElmer, Waltham, USA). Besides, to examine the ex vivo distribution of Hy‐M‐Exo, mice were euthanized at predetermined time intervals post‐injection, and the PLNs and ILNs were excised for ex vivo imaging. Meanwhile, the organs were obtained for *ex vivo* imaging.

For distribution of nanovaccines in LNs, DiD‐labeled TEX, DCMV, or Hy‐M‐Exo were administrated in the footpad of mice's left hind leg. After 24 h, the leftward PLNs were obtained, embedded with O.C.T medium, cryostat‐sectioned (8 µm), and fixed with cold acetone for 10 min. After being washed with PBS three times, the sections were blocked with 10% goat serum at 37 °C for 2 h. Then, slices were incubated with AF488‐anti‐CD3*ε* and rabbit‐anti‐mouse CCL21 overnight at 4°C. The slides were then washed five times with PBS and incubated with fluorescent goat‐anti‐rabbit secondary antibody at 37°C for 1 h. After being washed five times, tissue slices were mounted for observation on the CLSM.

### In Vivo Immunization with Hy‐M‐Exo

6–8‐week‐old female C3H mice (*n* = 5 for each group) were vaccinated with 100 µL PBS, TEX (50 µg protein), DCMV (50 µg protein), Hy‐Exo (50 µg protein), or Hy‐M‐Exo (50 µg protein, 5 µg MPLA) in the footpad of mice left hind leg, once a week for 3 weeks (Figure S6A, Supporting Information). Three days post the last vaccination, all mice were euthanized and the PLNs and ILNs were obtained and dissociated into single‐cell suspension, for analysis of the activated T cells using flow cytometry. The spleens were also harvested, dissociated into single‐cell suspension, cultured, and restimulated, for analysis of the CTLs using flow cytometry and for IFN‐*γ* ELISPOT detection. The peripheral blood was collected for detecting the levels of cytokines IL2, IFN‐*γ*, and TNF‐*α* by ELISA

### Elispot Assay

IFN‐*γ* secretion by splenocytes was conducted according to the manufacturer's protocol provided by DAKEWE (Shenzhen, China). The precoated IFN‐*γ* kit was activated by rinsing with 70% ethanol. The cell preparation was brought into the wells of the ELISPOT plates with a cell number of 2 × 10^5^ per well. Then the plates were incubated for 24 h, then shaken to remove cells, and rinsed with PBS two times. Then the plate was incubated with cold deionized water at 4 °C for 10 min to remove the rest of the cells and the biotinylated detection antibody was added to each well. The plate was incubated for 1 h at 37 °C and the solution was poured out. The plate was washed five times with PBS. Next, the streptavidin‐HRP conjugate was added and further incubated for 1 h at room temperature (RT). Then, the plate was emptied and both sides of the PVDF membranes were washed with water. The AEC solution was added into each well and incubated for 30 min at RT in the dark. The reaction was terminated by removing the solution and the plate was washed thoroughly with water and air‐dried at RT in dark. Count spots by use of an immunospot image analyzer.

### Therapeutic Study

For the therapeutic study, 6–8 week‐old female C3H mice were inoculated with 5 × 10^5^ SCCVII cells subcutaneously at the right flank at day 0. Tumor size and body weight were measured every 3 days, and the tumor volume was calculated as 0.5 × length × width^2^. When the tumor volume approached nearly 50 mm^3^, mice were vaccinated once a week with different vaccine formulations for three weeks. When the tumor volume of the PBS group exceeded 2000 mm^3^, all mice were euthanized and tumor masses were excised for analysis of the T‐cell subsets by flow cytometry. Major organs (heart, liver, spleen, lung, and kidney) were collected for H&E staining.

### Cell Viability Assay

In vitro cell viability of Hy‐M‐Exo was evaluated by the CCK‐8 assay. Briefly, 1 × 10^4^ DC2.4 cells or L929 cells were placed in 96‐well plates. After 12 h, cells were incubated with a fresh culture medium containing Hy‐M‐Exo at different concentrations for 24 h. CCK‐8 was added and incubated for another 3 h. Afterward, absorbance was recorded at 450 nm.

### Hemolysis Assay

Mouse whole blood was centrifuged at 3000 rpm for 5 min and washed three times with PBS to get pure RBCs. Different amounts of Hy‐M‐Exo were added into RBC suspension at final concentrations of 20, 50, 100, and 200 µg mL^−1^ and incubated at 37 °C for 2 h. RBC suspension mixed with pure water served as a positive control. The samples were centrifuged at 10000 g for 5 min and the absorbance of supernatants was measured at 540 nm.

### Statistical Analysis

All data were displayed in mean ±  standard error of mean deviation (SEM). Statistical analysis was performed using Prism 8.0 (GraphPad, USA). Unpared two‐tailed Student's *t*‐test was used for comparison between the two groups. One‐way ANOVA with Dunnett's post hoc analysis was performed for multiple‐group analysis. Statistical significance was present as *p < 0.05; **p < 0.01, ***p < 0.001, and ****p < 0.0001.

## Conflict of Interest

The authors declare no conflict of interest.

## Author Contributions

J.X., H.L., and T.W. contributed equally to this work. J.X.: conducted the main experiments; H.L.: assisted with partial experiments and supervision; T.W.: assisted with partial experiments; Z.W. and H.C.: assisted with materials characterization; Z.Y. and L.L.: supplied particle preparation; S.Y.S.G., and L.Y.: assisted with data analysis; K.L., J.L., and X.L.: supplied discussion; L.L.: supplied supervision; G.L. and Y.C.: supplied supervision and wrote the manuscript; Y.L.: designed, supervised all the experiments and wrote the manuscript.

## Supporting information

Supporting Information

## Data Availability

The data that support the findings of this study are available from the corresponding author upon reasonable request.
