# Supplementary material for: CCR7 Mediated Mimetic Dendritic Cell Vaccine Homing in Lymph Node for Head and Neck Squamous Cell Carcinoma Therapy
Source: Adv Sci (Weinh). 2023 Apr 24;10(17):2207017. doi: 10.1002/advs.202207017 (PMC10265089; doi:10.1002/advs.202207017)
Supplement: Supplementary file 1 — Supporting Information [file ADVS-10-2207017-s001.pdf]

ADVANCED SCIENCE
Open Access

Supporting Information

for *Adv. Sci.*, DOI 10.1002/advs.202207017

CCR7 Mediated Mimetic Dendritic Cell Vaccine Homing in Lymph Node for Head and Neck Squamous Cell Carcinoma Therapy

Jiabin Xu, Hong Liu, Tao Wang, Zhenfu Wen, Haolin Chen, Zeyu Yang, Liyan Li, Shan Yu, Siyong Gao, Le Yang, Kan Li, Jingyuan Li, Xiang Li, Lixin Liu, Guiqing Liao*, Yongming Chen* and Yujie Liang**

Supplementary materials

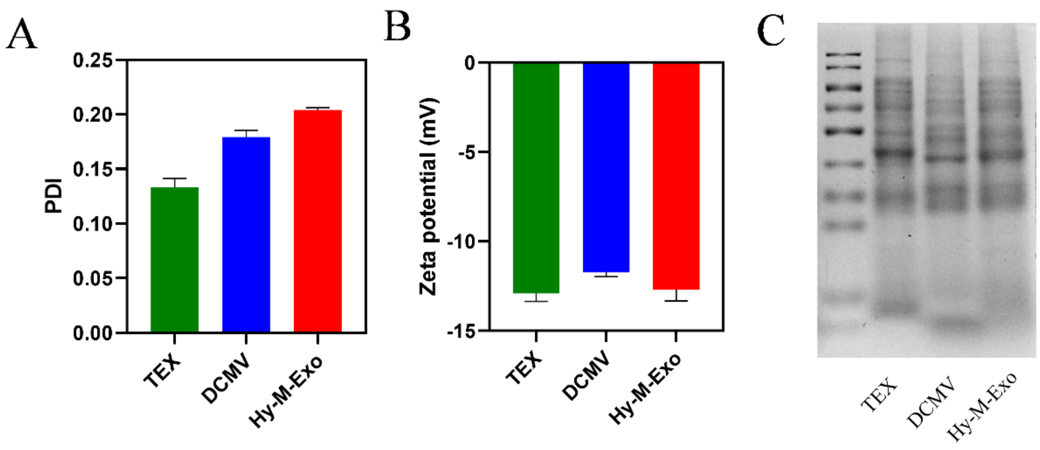

Figure S1. Characterization of various nanovesicles. (A) Polymer dispersity index (PDI) and (B) zeta potential measured by DLS. Data were means ± standard error of mean deviation (SEM) (*n*= 3). (C) Total protein of various nanovesicles characterized by Coomassie blue staining.

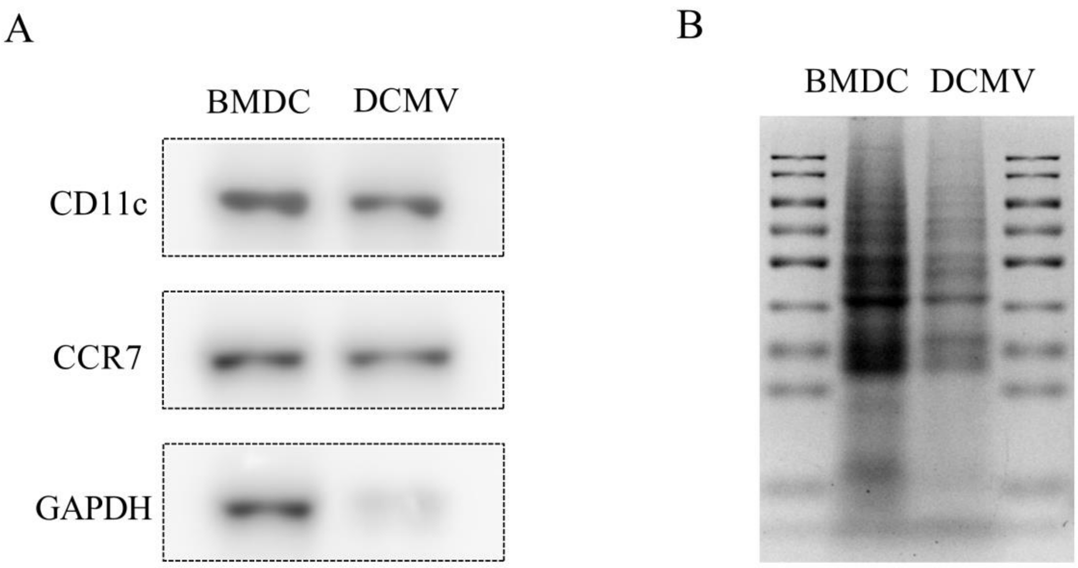

Figure S2. Protein markers analysis of DCMV characterized by (A) western blot and (B) Coomassie blue staining.

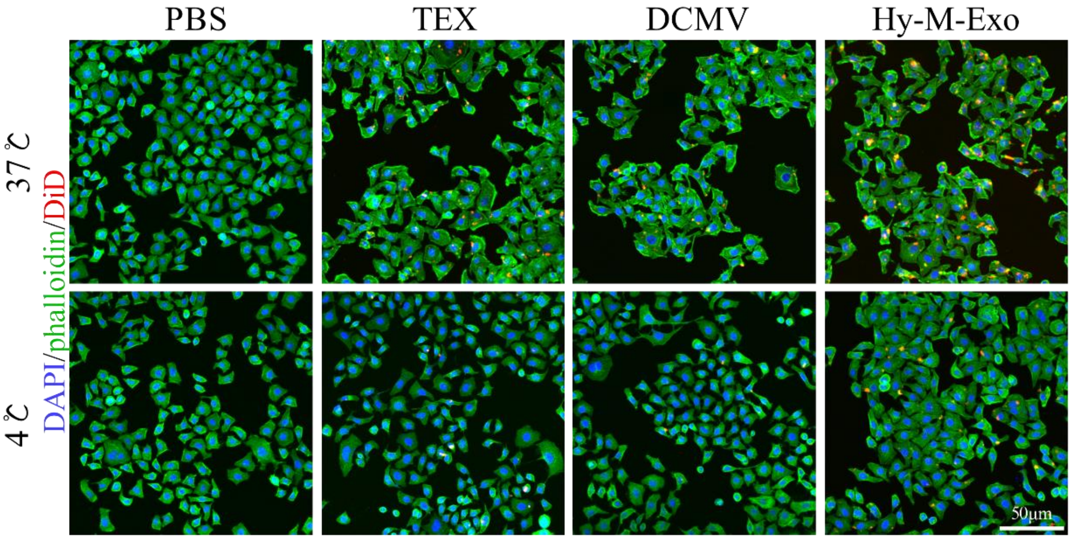

Figure S3. Uptake of Hy-M-Exo by DC2.4 cells is partly energy-independent. DC2.4 cells were cultured with DiD-labeled nanovesicles (50 μg mL^{-1}) at 37 °C or 4 °C for 4 h and the uptake was determined by confocal microscopy.

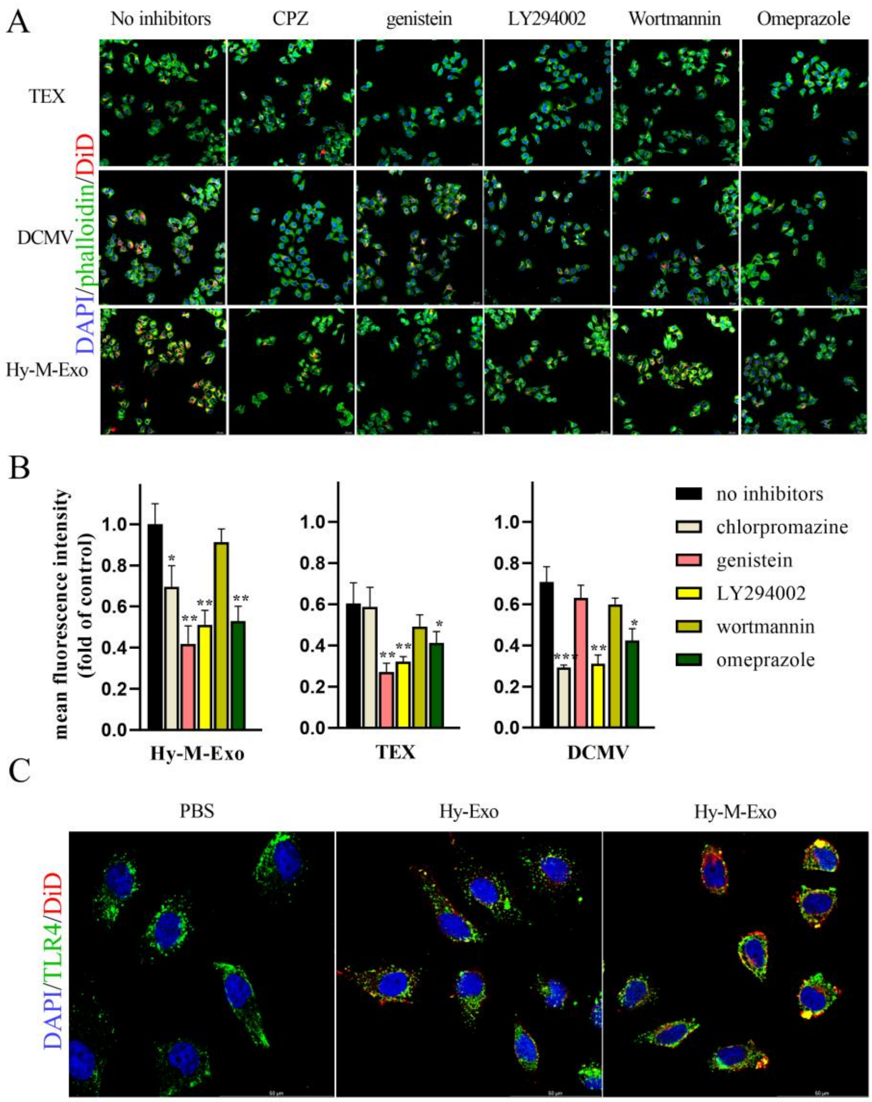

Figure S4. Uptake of Hy-M-Exo is mediated by the synergy of multiple pathways. DC2.4 cells were pretreated with different kinds of inhibitors for 30 min and then incubated with DiD-labeled nanovesicles for another 4 h in the presence of the indicated inhibitors. (A) The uptake was visualized by confocal microscopy, scale bar: 50 μm. (B) The mean fluorescence intensity was measured by flow cytometry. Data were means ± SEM (n= 3). No inhibitors group in different nanovesicles groups was used as the control. Statistical analysis was performed using unpaired two-tailed Student's t-test. *p<0.05, **p<0.01 and ***p <0.001. DC2.4 cells were cultured with DiD-labeled Hy-Exo and Hy-M-Exo (50 μg mL^{-1}) for 30 min, fixed with 4% paraformaldehyde for 10 min, and incubated with anti-TLR4 antibody for 1 h. (C) The uptake was determined by confocal microscopy, scale bar: 50 μm.

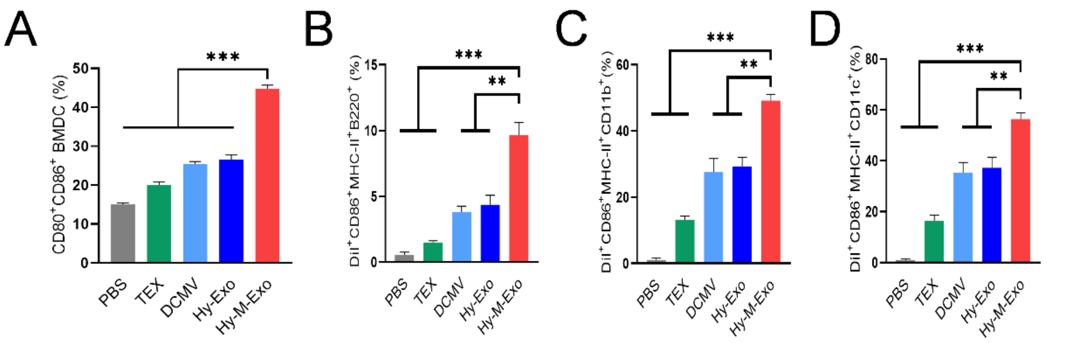

Figure S5. Activation of APCs by Hy-M-Exo. (A) Quantitative analysis of mature ($CD80^{+}CD86^{+}$) BMDCs after incubation with various nanovesicles for 24 h. (B-D) Maturation of APCs in draining LNs. C3H mice were injected with DiI-labeled nanovesicles. Popliteal and inguinal LNs were harvested for flow cytometry at 12h after injection. Data were presented as the mean ± SEM (n = 5). Statistical analysis was performed using unpaired two-tailed Student’s t-test. **$p < 0.01$, ***$p < 0.001$.

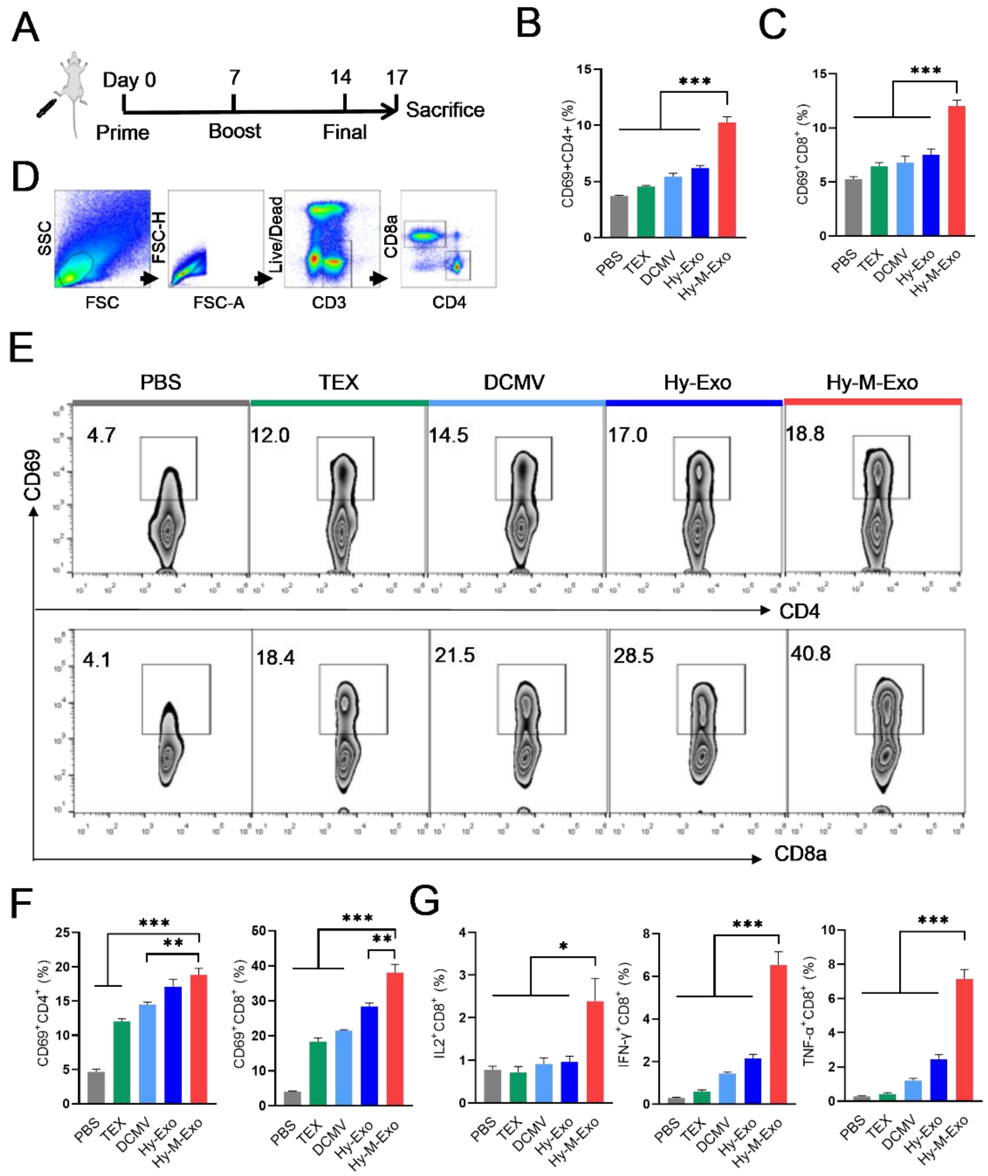

Figure S6. Hy-M-Exo induced T cell activation *in vivo*. (A) Schematic image of the immunostimulation study in C3H mice. Draining LNs (dLNs) and spleens were harvested 3 days after the final vaccination for flow cytometry. Quantitative analysis of (B) $CD69^{+}CD4^{+}$ and (C) $CD69^{+}CD8^{+}$ T cells in dLNs. (D) Representative gating strategy for detecting the $CD4^{+}$ and $CD8^{+}$ T cells by flow cytometry. (E) Representative cytometry images and (F) quantitative analysis of $CD69^{+}CD4^{+}$ and $CD69^{+}CD8^{+}$ T cells in different groups. (G) Peripheral serum levels of cytokines IL2, IFN-γ, and TNF-α detected with ELISA. Data were presented as the mean ± SEM (n = 5). Statistical analysis was performed using unpaired two-tailed Student's t-test. *$p < 0.05$, **$p < 0.01$, ***$p < 0.001$.

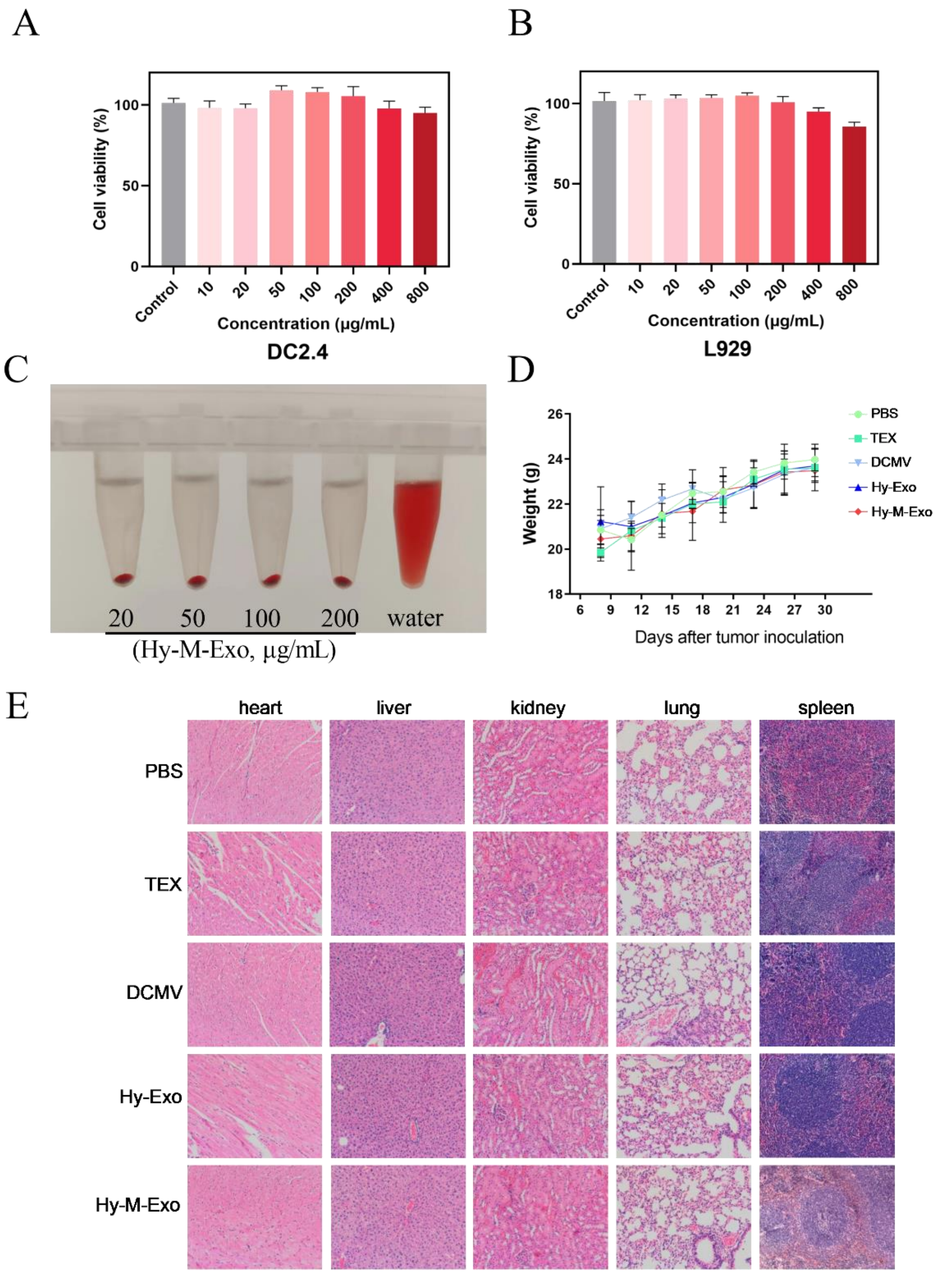

Figure S7. Biosafety evaluation of Hy-M-Exo. Cell viability of (A) DC2.4 and (B) L929 cells incubated with different concentrations of Hy-M-Exo for 24 h detected by CCK-8 (n = 3). (C) Representative image of RBCs incubated with Hy-M-Exo at different concentrations for 2 h. (D) Body weight of the tumor-bearing mice following tumor inoculation and various nanovesicles (n = 4). (E) Representative H&E staining images of heart, liver, kidney, lung, and spleen from mice treated with various nanovesicles. Data were presented as the mean ± SEM.
